# Determinants of technology adoption among healthcare professionals at Mogadishu hospitals using an extended UTAUT model

**DOI:** 10.3389/fdgth.2026.1819158

**Published:** 2026-06-04

**Authors:** Omar Osman Haji Abdi, Mohamed Jama Mohamed, Mohamed Ali Osman, Abukar Mukhtar Omar, Abdirahman Ibrahim Abdi

**Affiliations:** 1 Graduate School, SIMAD University, Mogadishu, Somalia; 2 Faculty of Computing, SIMAD University, Mogadishu, Somalia; 3 Faculty of Education, SIMAD University, Mogadishu, Somalia

**Keywords:** healthcare, hospital management, information system, technology adoption, UTAUT model

## Abstract

The study used the expanded Unified Theory of Acceptance and usage of Technology (UTAUT) to identify the variables influencing the usage of technology by medical staff in hospitals in Mogadishu. This study aimed to identify the key factors influencing technology usage among hospital staff in Mogadishu, Somalia, using an expanded Unified Theory of Acceptance and Use of Technology (UTAUT) framework. A quantitative research design was employed, based on survey data collected from 470 hospital staff members across selected hospitals in Mogadishu. Structural equation modeling (SEM) was used to examine the relationships between variables and assess the validity of the measurement model. The findings show convergent validity and good indicator reliability across the majority of components, with outer loadings ranging from 0.742 to 0.897. Overall construct reliability and convergent validity were excellent, supporting its preservation, despite age and technological innovation did not significantly moderate the relationships between UTAUT factors and perceived usefulness, suggesting that these effects are consistent across different user groups. The results also showed the *p* values of the UTAUT factors are less than 0.05 which means that they significantly enhance perceived usefulness, but Age and technological innovation, had no statistically significant moderating effects on the associations between UTAUT predictors and perceived usefulness, suggesting that the effects were consistent across user groups. The study offers empirical insights for the use of technology in hospital settings and helps validate an expanded UTAUT paradigm in healthcare. Future studies should include other moderating factors, incorporate longitudinal data, and adjust technological innovation criteria to improve model robustness

## Introduction

Hospital Management Information System is a formal technique that supports management activities. Provision of high-quality services must be prioritized by the health sector in all countries to improve patient care, as recent realizations by governments and healthcare organizations have highlighted the crucial role that technology plays in reaching this objective (1, 2). According to Keskinocak & Savva (3) sustainable development requires fostering well-being at all ages and guaranteeing healthy lives. Across many countries, information and communication technology is recognized as a critical tool for health care delivery and public health. A study conducted by Aljohani & Chandran (2) revealed that countries, like Malaysia, Thailand, China, and India, have also been incorporating technological advancements in healthcare services, in order to allow medical professionals to exploit technological breakthroughs. Health information systems were also widely used by healthcare practitioners in the United Kingdom, Canada, the United States, Germany, and the Netherlands (4).

Numerous investigations conducted on healthcare facilities have revealed that excellent record-keeping by patients regarding their medical background, present state of health, and course of treatment support both health professionals and management activities by automating the efficient planning, controlling, and decision-making processes (1, 5).

Despite having 12% of the world's population, Sub-Saharan Africa (SSA) employs 3.5% of the health workforce worldwide to manage a disproportionate 27% of the world's illness burden (6). According to Enos & Basaza-Ejiri (4), the usage of hospital management systems is low in various developing countries, including Ethiopia, Nigeria, and Egypt, and they all face common challenges such as a lack of trained human and material resources. For this reason, the information system's acceptance and use are directly correlated with the knowledge and proficiency of its users (1).

Even though the growing body of literature on Hospital Management Systems (HMS), the extant knowledge, particularly in unstable and post-conflict situations such as Somalia, is insufficient. Furthermore, past research has mostly concentrated on the direct determinants that drive technology adoption, with little attention given to the moderating mechanisms that can influence these connections.

Improving hospital operations and patient care requires the use of hospital management systems (HMS). Therefore, hospitals must have a framework in place to assist them in fulfilling their responsibilities as health-care providers (7). A study conducted by Enos & Basaza-Ejiri (4); Aljohani & Chandran (2) revealed that obstacles hindering the implementation of hospital management system include inadequate infrastructure, a lack of appropriate policies and regulations, a lack of financial resources, weak governance, and a shortage of competent labor which must be met in order to install new hospital management system. Thus, research on technology adoption aims to elucidate the elements that influence the success or failure of information systems and technologies (8).

The inclusion of technological innovation as a moderating variable in this study is supported by innovation diffusion theory and the extended Unified Theory of Acceptance and Use of Technology (UTAUT2), which recommend that the impact of core determinants such as performance expectancy, effort expectancy, and facilitating conditions may differ depending on an organization's level of technological advancement. Previous research has found that firms with higher levels of technical innovation are more likely to improve user views of system usefulness and ease of use, hence enhancing adoption outcomes (9).

As a result, the analysis in this study will look into the elements that influence hospital management systems, utilizing the extended UTAUT model to assess health workers' acceptability levels. This is due to the fact that healthcare organizations around the globe are making more investments in information technology (IT), which has led to the realization that user acceptance is an essential part of the deployment and management of technology (10). The study was conducted at Mogadishu hospitals because it is the most populated city in Somalia and has a variety of healthcare facilities that are gradually embracing digital technologies, giving it an ideal location to investigate the dynamics of HMS acceptability.

## UTAUT model constructs and research hypotheses

The Unified Theory of Acceptance and Use of Technology (UTAUT) model incorporates and builds upon eight earlier models and/or ideas pertaining to the adoption and use of technology (11). The models reviewed and integrated into UTAUT include Technological Acceptance Model (TAM, 1989), Motivational Model (MM, 1992), Model of PC Utilization (MPCU 1991), Theory of Planned Behavior (TPB, 1991), and Combined TAM and TPB (C-TAM-TPB, 1995) (12). UTAUT was first conceptualized, improved, and tested experimentally in 2003 (13). The criticism that TAM was inadequate in explaining user behavior and the challenge of enhancing the model's predictive ability led to the development of the successful UTAUT model (1). Technology acceptance models, such as TAM and UTAUT, have been used in a number of studies to investigate technology adoption in healthcare institutions (12). However, the UTAUT model has been employed in just a few health facility studies conducted in developing countries. The model is up to date and aligned with advancements in technology. The UTAUT model's four primary components encouraged researchers to use. The extended AUTAUT Model in this study has four independent variables which are: Performance Expectance (PE), Effort Expectancy (EE).Social Influence (SI) and Facilitating Conditions (FC); the UTAUT Model also has three dependent variables, Behavioral Intention (BI) and Behavioral Use of technology (BU) and two moderators Age and Technological Innovation (TI).

## Performance expectancy

The latent variables include Performance expectation (PE) is the extent to which an individual believes that using a certain system would benefit him or her more and improve work performance. Due to the widespread adoption of technology in various industries, it has recently become a part of daily life (14). According to a number of studies conducted by Adirinekso et al. (15) reveled that performance expectation has a significant role in influencing the intentional use of technology. The extent to which a person thinks that utilizing a certain system or technology would improve performance by making daily tasks easier is known as performance expectation (16). In the context of technology adoption and usage, performance expectation is a reliable predictor of behavioral intention (17). This pertains to a person's belief and assurance that incorporating information and communication technology (ICT) into their everyday work processes and professional activities may greatly improve their job performance and boost productivity. In other words, healthcare professionals will utilize the technology if they believe it would improve their performance. Technological innovation and age act as moderators of the relationship between Performance Expectance and Perceived Usefulness.

H1: Performance Expectancy has a positive effect on Perceived Usefulness.

H2: The relationship between Performance Expectancy and Perceived Usefulness is moderated by Technological Innovation.

H3: The relationship between Performance Expectancy and Perceived Usefulness is moderated by the age.

## Effort expectancy

Effort Expectancy (EE) is the degree to which people use a particular technology with ease. Effort expectation (EE) is a measure of how easy technologies are to use, and prior studies have shown that EE is a strong predictor of technology usage intention (16–18) and UTAUT has determined that it is a significant element influencing behavioral intention to embrace the technology (19). In the UTAUT paradigm, Effort Expectancy (EE)—also known as Perceived Ease of Use in the TAM model—represents the user's subjective expectation that Telehealth would be simple to use (20). Rahi et al. (21) argues that positive performance results and the system's simplicity of use are two factors that affect a person's inclination to utilize a particular system According to Aljohani & Chandran (2), Effort Expectancy is a key component of the UTAUT model and has been found to have a significant impact in various research. It has been acknowledged as a major influence on the adoption of mobile health tracking systems, phone-based e-health services, and medical decision support systems, emphasizing its significance in determining user acceptability and interaction with health technology. The link between perceived usefulness and Easy Expectance are moderated by age and technological innovation.

H4: Effort Expectancy has a positive influence on the Perceived Usefulness.

H5: The relationship between Effort Expectancy and Perceived usefulness is moderated by the age.

H6: The relationship between Effort Expectancy and Perceived usefulness is moderated by Technological Innovation.

## Social influence (SI)

Social Influence (SI) is the extent to which other people's opinions influence how people behave when using new technology (22).This means that social influence is one of the fundamental concepts of the UTAUT model, which describes how much a person feels that significant others think they should adopt the new system (2) and Numerous research have been carried out to examine how social influence affects people's desire to use technology (23). In the context of telemedicine, it is defined as the degree to which the patient feels that the recommendations of others are relevant for embracing telemedicine health services (24). Researchers have thoroughly investigated the concept of social influence and demonstrated its impact on shaping individuals' intentions to use and accept various technological innovations, such as electronic library services, especially in the early stages of development when users lack information and experience with the new technology (9). Thus, numerous studies conducted in a variety of sectors, including Healthcare Information Technology (HIT), have shown that social influence has little bearing on people's intentions to utilize IT and its applications (25). Age and technical development moderate the relationship between perceived usefulness and Social Influence.

H7: Social Influence has a positive influence on the Perceived Usefulness.

H8: The relationship between Social Influence and Perceived Usefulness is moderated by the age.

H9: The relationship between Social Influence and Perceived Usefulness is moderated by Technological Innovation.

## Facilitating conditions (FC)

The degree to which people believe there is enough organizational and technical assistance to help them accept and use technology is referred to as facilitating conditions (26). As the Isa et al. (27) argues that facilitating Conditions relate to the technical aid supplied by the external environment, which includes organizational and technological support such as computer hardware, software, and system operating assistance in order for the system to be used effectively. Several studies have shown that Facilitating Conditions play an important and beneficial influence in encouraging the usage of information technology, particularly health information systems (25). In contrast to this (23) found that facilitating condition has insignificant effect on behavioral use of technology. Similarly, a study conducted by Venkatesh, stated that enabling conditions defined as the availability of relevant resources and support in using technology do not increase behavioral intention, but they have a significant influence on actual use behavior (28), thus, the UTAUT model, four essential variables performance expectation, effort expectancy, social influence, and enabling conditions have a direct impact on behavioral intentions, while age, gender, experience, and voluntariness of usage serve as moderators (22). The relationship between Facilitating condition and perceived usefulness are moderated by age and Technological Innovation.

H10: Facilitating conditions will have a positive influence on Perceived Usefulness.

H12: The relationship between Facilitating conditions and Perceived Usefulness is moderated by age.

H12: The relationship between Facilitating conditions and Perceived Usefulness is moderated by Technological Innovation.

## Perceived usefulness

The degree to which an individual thinks that utilizing a certain system will enhance his performance at work is known as usefulness (19, 29). Therefore, a high perceived usefulness of technology might boost individual trust, which will reinforce its adoption (30, 31). Another study conducted by (32) revealed that behavioral intention to utilize the technology was highly impacted by perceived usefulness. This means that technology improves work performance when healthcare procedures are digitalized, which influences their behavior.

H13: Perceived Usefulness will have a positive influence Behavioral Intention.

## Behavioral intention

**Figure 1:**
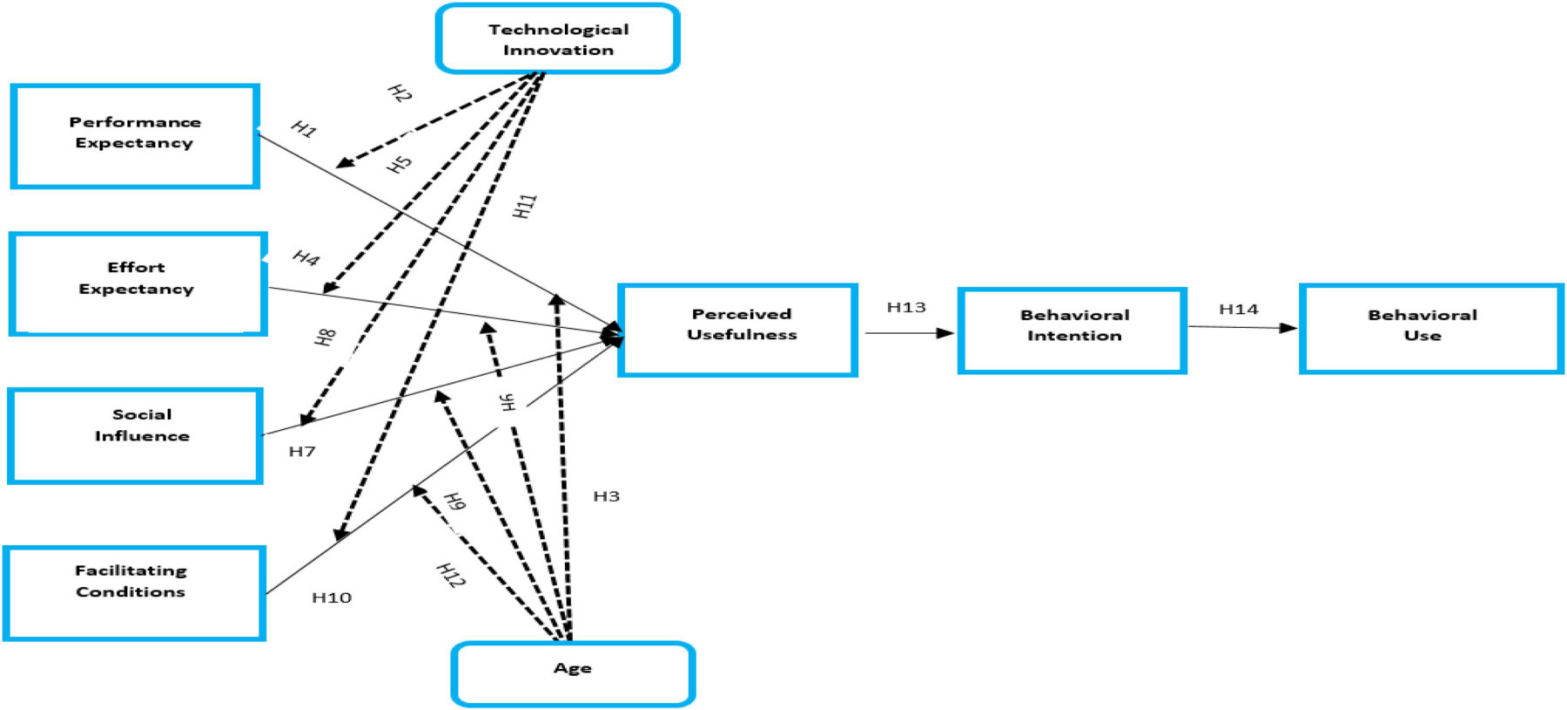
UTAUT model. Adapted from Venkatesh et al. (43).

A person's desire or readiness to utilize a technological system in the future is referred to as behavioral intention. According to some research in the literature, behavioral intention has a significant and favorable impact on embracing or utilizing new technologies (2). In contrast to this Dwivedi et al. (33) revealed that behavioral intention may not have a particularly significant or consistent influence on use behavior. The research proposed model is show in Figure 1 below.

H14: Behavioral Intention will have a positive influence on Behavioral Use on Technology.

## Methodology

The study's main goal is to use extended Unified Theory of Acceptance and Use of Technology (UTAUT) model to examine the variables impacting the adoption of Health Management Information System (HMIS) technology in hospitals in Mogadishu, Somalia. The UTAUT model was selected by the researchers because of its inclusive nature, which allows them to take into account not only the technological elements of implementing data analytics methods but also the behavioral attitudes and readiness of healthcare personnel to accept them (34). The study expanded on the original UTAUT Model (performance expectancy, effort expectancy, social influence, and facilitating conditions) by including perceived value (Mediator), age, and technological innovation (Moderators), which contribute to how the main factors influence behavioral intention and actual use of HMIS technology among healthcare professionals.

This study gathered and analyzed quantitative data using a descriptive research design to evaluate (35) the adoption of Health Management Information System (HMIS) technology in hospitals in Mogadishu. The quantitative design enables statistical generalization of the results across hospitals in Mogadishu and is appropriate for assessing correlations between dimensions in the UTAUT model.

The questionnaire's measuring items were modified using validated scales found in the literature, especially from earlier research using the UTAUT and extended UTAUT models in information systems and healthcare settings. To guarantee contextual relevance to HMIS usage in Mogadishu hospitals, a few minor adjustments were implemented.

A systematic questionnaire was created utilizing the extended UTAUT constructs to gather primary data. There are two primary portions of the questionnaire. The demographic data of the respondents, such as gender, age, occupation, and years of experience, are recorded in the first part. Performance expectancy, effort expectancy, social influence, facilitated conditions, perceived value, behavioral intention, age, technological innovation, and HMIS usage behavior are all measured in the second phase of UTAUT. A five-point Likert scale, was used to rate each construct item.

Healthcare workers that actively utilize HMIS in Mogadishu's public and private hospitals, including physicians, nurses, health information officers, and administrative personnel, are among the target group. Purposive sampling was used because the study focuses on healthcare professionals who have firsthand experience with HMIS, making them the best respondents to provide pertinent and trustworthy data. However, it is acknowledged that the sample may not precisely reflect the entire population of healthcare personnel in Mogadishu, as purposive sampling may limit the data’ generalizability and introduce selection bias.

The nonprobability sampling strategies need judgment since individuals are chosen depending on their accessibility (36). The sample size of respondents was 470 to improve the statistical power and dependability of the findings.

The goals of the study were explained to each participant, and their voluntary participation was ensured via informed consent. No personally identifiable information was gathered, and respondents were guaranteed anonymity and confidentiality.

Data analysis was done using SPSS 27 for demographic information analysis and the Smart PLS 4.0 software program to assess the research model (13). Because Smart PLS is suitable for complicated models with high sample sizes and predictive analysis, its application is appropriate.

## Results

### Demographic information of the respondents

According to the demographics in Table 1 the male respondents constitute (60.2%), while the remaining 39.8% is female. Most of healthcare Professionals (45.96%) were between the ages of 31 and 40, followed by those between the ages of 20 and 30 (37.02%), with just 17.02% being older than 41. In terms of position, the sample is primarily made up of nurses (76.8%), with lower percentages of lab technicians (10%), physicians (9.1%), and administrative personnel (4.1%). Nearly half of healthcare professionals' job experience was between 4 to seven years (48.1%), while 34% had 1–3 years and 17.9% had 8 or more years. The most of participants are in the early to mid-stages of their careers.

**Table 1:** Demographics’ characteristics.

Gender	Frequency	Percent (%)
Male	283	60.2%
Female	187	39.8%
Total	**470**	**100%**
Age		
20–30	174	37.02%
31–40	216	45.96%
41 and above	80	17.02%
Total	**470**	**100%**
Position		
Doctor	43	9.1%
Nurse	361	76.8%
Lab technician	47	10%
Admin	19	4.1%
Total	470	**100%**
Work experience		
1–3	160	34%
4–7	226	48.1%
8 and above years	84	17.9%
Total	470	**100%**

Note: Bold values indicate total frequencies and percentages for each category.

### Measurement of the model

In recent years, structural equation modeling (SEM) has grown in popularity because it allows researchers to analyze the reliability and validity of multi-item construct assessments while also assessing structural model linkages (13).

**Figure 2:**
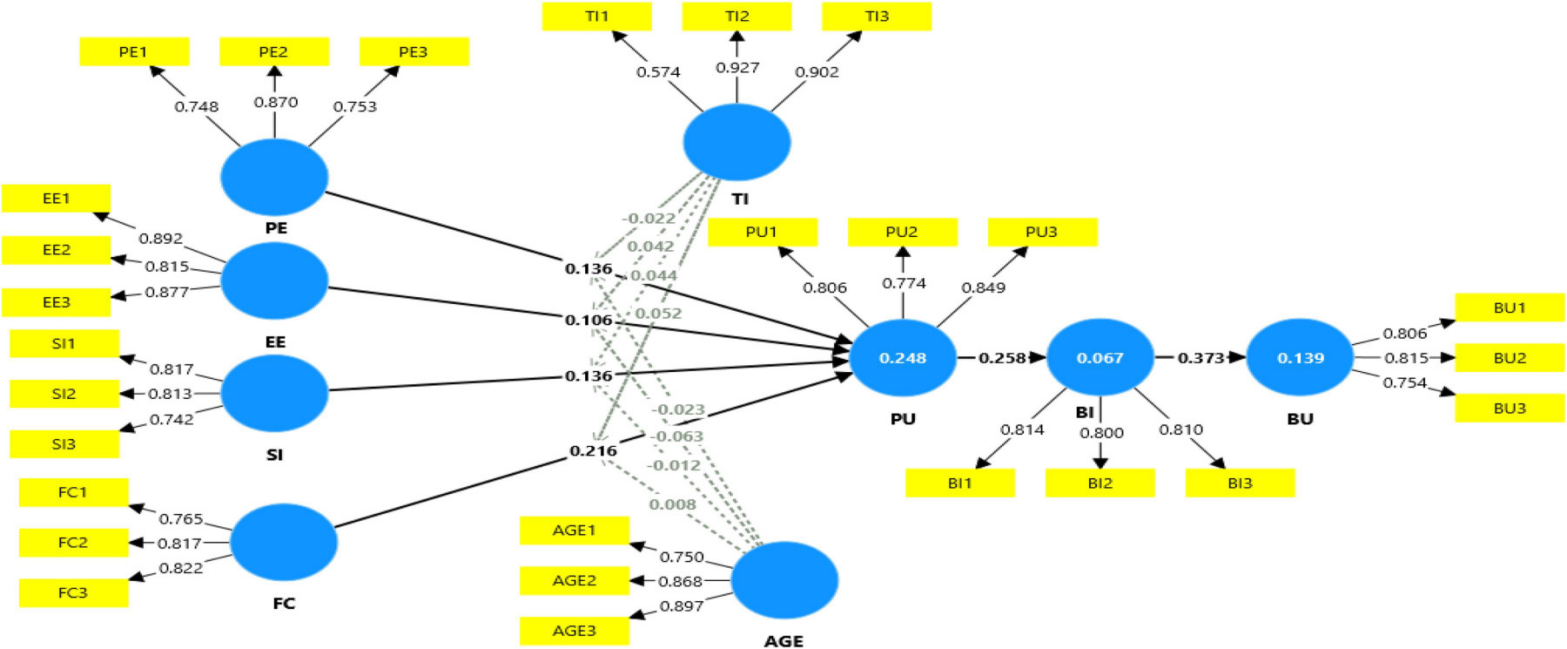
Research model.

The construct variables of the extended model are Performance Expectancy (PE), Effort Expectancy (EE), Social Influence (SI), Facilitating Conditions (FC) Technological Innovation (TI) (as a moderator) and Age (AGE) (as a moderator) with Perceived Usefulness **(**PU) and Behavioral Intention (BI) as a mediating constructs**,** while Behavioral Use (BU) as a predictor of the behavior. As shown in Figure 2, most indicator loadings exceed the commonly accepted threshold of 0.70, indicating adequate indicator reliability.

The measurement model assessment results, including Cronbach's alpha, composite reliability and average variance extracted (AVE) for each construct, are shown in Table 2. The outer loadings show that the majority of indicators load significantly on their corresponding structures, surpassing the suggested cutoff of 0.70. But one trust indicator (TI1) has a relatively low loading (0.574), indicating possible measurement problems and calling for additional convergent validity testing.

**Table 2:** Reliability assessment.

Construct	Items	Loading	Cronbach's alpha	CR	AVE
AGE	AGE1	0.750	0.792	0.878	0.707
	AGE2	0.868			
	AGE3	0.897			
Behavioral Intention	BI1	0.814	0.735	0.850	0.653
	BI2	0.800			
	BI3	0.810			
Behavioral Use	BU1	0.806	0.706	0.835	0.628
	BU2	0.815			
	BU3	0.754			
Effort Expectancy	EE1	0.892	0.827	0.896	0.743
	EE2	0.815			
	EE3	0.877			
Facilitating Conditions	FC1	0.765	0.722	0.844	0.643
	FC2	0.817			
	FC3	0.822			
Social Influence	SI1	0.742	0.701	0.834	0.626
	SI2	0.817			
	SI3	0.813			
Performance Expectancy	PE1	0.748	0.712	0.834	0.628
	PE2	0.870			
	PE3	0.753			
Perceived Usefulness	PU1	0.806	0.738	0.851	0.656
	PU2	0.774			
	PU3	0.849			
Technological Innovation	TI1	0.574	0.736	0.853	0.667
	TI2	0.927			
	TI3	0.902			

All constructions support the reliability of internal consistency. The required criterion of 0.70 is exceeded by Cronbach's alpha values, which vary from 0.701 to 0.827 and indicate excellent internal consistency. The measurement scales' reliability is further confirmed by the composite reliability values for both (0.706–0.837) and (0.834–0.896), which are higher than the permissible threshold of 0.70.

Additionally, convergent validity is proven. Every construct has an AVE value above 0.50 (range from 0.626 to 0.743), meaning that more than half of the variance of its indicators can be explained by each construct. With the highest Cronbach's alpha (0.827), composite reliability (0.896), and AVE (0.743) among the constructs, Effort Expectancy (EE) exhibits the best measurement qualities. The suggested reliability and validity standards are likewise met by all other constructions, including AGE, BI, BU, FC, PE, PU, SI, and TI.

Significantly, these substantial reliability and validity indicators raise confidence in the interpretation of the path relationships by indicating that the measurement model is sound and appropriate for structural analysis.

### Discriminant validity

The evaluation of discriminant validity using the Fornell-Larcker criterion is presented in Table 3. Two approaches for assessing discriminating validity are item loadings to variable correlations and the ratio of each variable's square root of the AVE to its correlations with all other variables (8). The square roots of AVE for each construct are shown by the diagonal elements (varying from 0.791 to 0.862) in the table, whilst the inter-construct correlations are indicated by the off-diagonal elements. Each concept shares more variance with its own indicators than with other constructs, as evidenced by the diagonal values always exceeding the equivalent correlations with other constructs.

**Table 3:** Discriminant validity.

Constructs	AGE	BI	BU	EE	FC	PE	PU	SI	TI
AGE	**0**.**841**								
BI	0.359	**0**.**808**							
BU	0.222	0.373	**0**.**792**						
EE	0.084	0.152	0.098	**0**.**862**					
FC	0.188	0.242	0.343	0.026	**0**.**802**				
PE	0.196	0.240	0.302	0.012	0.276	**0**.**792**			
PU	0.240	0.258	0.297	0.148	0.351	0.296	**0**.**810**		
SI	0.219	0.288	0.282	0.046	0.271	0.303	0.305	**0**.**791**	
TI	0.190	0.283	0.525	0.165	0.237	0.231	0.283	0.314	**0.817**

Note: Diagonal values in bold represent the square root of the Average Variance Extracted (AVE) for each construct. BI, behavioral intention; BU, behavioral use; EE, effort expectancy; FC, facilitating conditions; PE, performance expectancy; PU, perceived usefulness; SI, social influence; TI, technology intention.

This finding ensures that the observed structural relationships reflect true theoretical associations rather than measurement overlap, and it alleviates concerns about multicollinearity by establishing the constructs' empirical distinctness.

### Results of path analysis

Perceived usefulness (PU) is significantly improved by Effort Expectancy (EE → PU) (*β* = 0.106, *p* = 0.012), Facilitating Conditions (FC → PU) (*β* = 0.216, *p* < 0.001), Performance Expectancy (PE → PU) (*β* = 0.136, *p* = 0.005) and Social Influence (SI → PU) (*β* = 0.136, *p* = 0.008). In comparison to the comparatively smaller effects of EE (*β* = 0.106), PE (*β* = 0.136), and SI (*β* = 0.136), Facilitating Conditions (*β* = 0.216) has the largest impact on Perceived Usefulness. This implies that individual judgments of ease of use or social pressure are less important than external support factors. The hypothesis is deemed acceptable if the T-statistic findings fall outside of the range of −1.96 to 1.96 (37). The model's strongest and most significant connection is between behavioral intention and actual usage (*β* = 0.373). In keeping with UTAUT theory, this highlights the importance of purpose as a significant driver of real-world system utilization.

On the other hand, low t-values and *p*-values significantly above 0.05 show that all moderation effects involving age and technological innovation are statistically insignificant. Age does not significantly change the associations between these predictors and perceived usefulness, according to interaction effects like AGE —–FC → PU (*p* = 0.890), AGE —–SI → PU (*p* = 0.807), AGE—-EE → PU (*p* = 0.234), and AGE —–PE → PU (*p* = 0.644). Technological innovation does not serve as a substantial moderator in the model, as seen by the insignificance of interactions involving Technological Innovation, such as TI—- SI, TI —–EE, TI—— FC, and TI——–PE (*p* > 0.30). According to (38) the hypothesis is deemed invalid or rejected if the T-statistic findings fall between −1.96 and 1.96.

The associations between the moderating factors and the UTAUT model constructs, which illustrate their moderating effects, are represented by the dotted lines in Table 4, whereas the solid lines indicate the direct correlations between the remaining model parts.

**Table 4:** Hypothesis testing.

Hypothesized paths	Original sample (O)	Sample mean (M)	Standard deviation (STDEV)	T statistics (|O/STDEV|)	*P* values
BI→BU	0.373	0.379	0.046	8.107	0.000
EE→PU	0.106	0.111	0.042	2.515	0.012
FC→PU	0.216	0.214	0.050	4.349	0.000
PE→PU	0.136	0.136	0.049	2.784	0.005
PU→BI	0.258	0.261	0.052	4.943	0.000
SI→PU	0.136	0.141	0.051	2.656	0.008
AGE―FC→PU	0.008	0.013	0.061	0.138	0.890
AGE―SI→PU	−0.012	−0.011	0.049	0.245	0.807
AGE―EE→PU	−0.063	−0.061	0.053	1.189	0.234
AGE―PE→PU	−0.023	−0.022	0.051	0.463	0.644
TI―SI→PU	0.044	0.042	0.053	0.830	0.407
TI―EE→PU	0.042	0.042	0.045	0.937	0.349
TI―FC→PU	0.052	0.053	0.053	0.977	0.329
TI―PE→PU	−0.022	−0.022	0.050	0.432	0.666

The relationships between key variables and perceived usefulness are consistent across age groups and levels of technological innovation, indicating that there are no significant moderating effects. From a critical standpoint, the lack of significance of these interaction effects may suggest that the associations between fundamental UTAUT-related variables and perceived value are consistent across age groups and levels of technological innovation.

## Discussion

This study aimed to assess the factors influencing users' perceived usefulness and behavioral intention toward technology adoption as well as moderating effects of age and technological innovation within the UTAUT framework. Structural equation modeling (SEM) has acquired significant attention in recent years and well suited to research with small sample sizes due to its capacity to concurrently analyze the reliability and validity of measurement models (13). This study employed indicator loadings, Cronbach's alpha, composite reliability (CR), and average variance extracted (AVE) to evaluate the measurement model.

Across all structures, the outer loading findings showed typically good indication reliability. Most indicators load over the suggested threshold of 0.70, suggesting that they appropriately reflect their respective latent variables. In particular, loadings for AGE, Behavioral Intention (BI), Behavioral Use (BU), Effort Expectancy (EE), Facilitating Conditions (FC), Social Influence (SI), Performance Expectancy (PE), and Perceived Usefulness (PU) range from 0.742 to 0.897, indicating strong convergent validity and satisfactory to high indicator reliability. These findings support the model's strength, as they are consistent with previous UTAUT-based studies that discovered similar good measurement features for core variables such as performance expectancy and effort expectancy.

Effort Expectancy and Behavioral Intention are particularly well measured, with all indicators exceeding 0.80, suggesting that the items are highly representative of their constructs. These results support the robustness of the measurement instruments used for these variables.

The Technological Innovation construct is an exception, with item TI1 having a loading of 0.574, which is lower than the suggested cutoff of 0.70. According to Alam et al. (39) the accuracy technological innovation in forecasting healthcare technology uptake is questionable, particularly in developing nations. In Africa ICT-supported health care services are relatively recent word in the field of medicine (12).

This raises the possibility of measuring issues such conceptual misalignment with the construct domain or item ambiguity. Nevertheless, the design still obtains good composite reliability and AVE values, while the remaining indicators (TI2 and TI3) load extremely significantly. Hair et al. (40) revealed that if overall construct reliability and convergent validity are satisfactory, as they are in this case, indicators with loadings of 0.40 to 0.70 may be retained.

Cronbach's alpha and composite reliability were also used to evaluate internal consistency reliability. With scores ranging from 0.701 to 0.827, all structures show high to exceptional internal consistency and above the minimum suggested criterion of 0.70 for Cronbach's alpha. The degree to which indicators within a construct are positively associated and assess the same underlying notion is shown by Cronbach's alpha (4). These findings support the measurement model's statistical adequacy and are consistent with earlier empirical research that validates UTAUT constructs in healthcare settings, indicating that the model maintains its explanatory value even in situations with limited resources. Similar results regarding the validity and reliability of measurement models have been reported by Aljohani & Chandran (2); Mohamed & Hassan (13); Sharifian et al. (10).

Composite reliability (CR) values are extra confirmation of the reliability. The CR values are all significantly higher than 0.70, ranging from 0.834 to 0.896. As emphasized by Hair et al. (40) composite reliability is chosen over Cronbach's alpha in SEM because it accounts for differing indicator loadings, providing a more precise assessment of construct reliability.

The average variance extracted (AVE) was used to assess convergent validity. With values ranging from 0.626 to 0.743, all constructs satisfy the suggested criteria of AVE > 0.50, meaning that each construct accounts for more than half of the variation of its indicators. Vantissha et al. (41) revealed that the minimum AVE of 0.50 is advised.

In addition to an analysis of moderating effects by Age (AGE) and Technological Innovation (TI), the diagonal elements, which represent the square root of the path analysis results shown in Table 3, reveal significant direct effects of several predictors on Perceived Usefulness (PU) and behavioral outcomes. A study conducted by Jayaseelan et al. (11) revealed that the essential variables (EE), (SI), and (FC), as well as the moderating factors age, experience, and behavioral purpose, are significantly correlated. The effect age on facilitating conditions was also found to be higher in elderly personnel compared to young personnel (1). These results contradict some earlier research that found age to be a significant variable in models of technology adoption. This disparity implies that, in this context, contextual elements like system standardization, organizational support, and training may be more important in influencing user views than demographic traits.

The hypothesized mediation chain under the UTAUT paradigm, were Perceived Usefulness and Behavioral Intention lead to behavioral use of technology. Low t-values and high *p*-values significantly over the 0.05 threshold indicate that all investigated interaction variables involving Age and Technological Innovation as moderators on the PU routes are not significant. Nonetheless, hospitals are increasingly anticipating the use of the newest technology to enable adjustments that enhance the patient experience (42). According to the results, merely implementing new technologies is not enough; users' perceptions of these technologies' utility, usability, and support within their workplace must be equally prioritized.

## Conclusion

This study indicated that major UTAUT constructs Performance Expectancy, Effort Expectancy, Social Influence, and Facilitating Conditions, greatly impact users' Perceived Usefulness, which in turn drives Behavioral Intention and actual Behavioral Use. These relationships are unaffected by age or technological innovation, suggesting that the benefits are consistent across a range of user demographics and degrees of innovation.

The results may not be as generalizable as they may be because the sample was drawn from a specific population and geographic location. Second, Technological Innovation showed a comparatively low factor loading, suggesting possible flaws in the measurement of the construct. This implies that in order to guarantee greater construct reliability and validity in further research, measuring scales need be improved and validated.

The researchers suggest that hospitals should prioritize providing strong technical and institutional support to enhance users' understandings of technological efficacy and encourage adoption. Involving opinion leaders and social networks can increase perceived usefulness and adoption intentions even further. To eliminate obstacles to utilization, institutions should ensure that enabling characteristics such as responsive IT support and system dependability are consistently maintained.

## Data Availability

The raw data supporting the conclusions of this article will be made available by the authors, without undue reservation.
